# O.3.3-3 Impact and lessons learned from 18 Years of BIG – a project to promote physical activity among women in difficult life situations

**DOI:** 10.1093/eurpub/ckad133.158

**Published:** 2023-09-11

**Authors:** Annika Herbert-Maul, Karim Abu-Omar, Raluca Sommer, Alexandra Sauter, Heiko Ziemainz

**Affiliations:** Friedrich-Alexander University Erlangen-Nuremberg, Germany; Friedrich-Alexander University Erlangen-Nuremberg, Germany; Friedrich-Alexander University Erlangen-Nuremberg, Germany; University Regensburg, Germany; Friedrich-Alexander University Erlangen-Nuremberg, Germany

## Abstract

**Purpose:**

Since 2005, practitioners and researchers have developed the BIG-project (“Movement as investment in health”), a community-based participatory research (CBPR) project that aims to empower socially disadvantaged women to engage in physical activity (PA) and exercise. These women are often unemployed, single mothers or belong to an ethnic minority facing barriers to exercise such as high registration fees, no availability of childcare or culturally insensitive programs. To date, the project has been scaled to 23 communities. The long project duration and multiple project site provide an excellent opportunity to investigate the long-term impacts of CBPR on PA promotion. The follow-up study of BIG (NU-BIG) examines the long-term effects on individual and structural levels at the project sites.

**Methods:**

NU-BIG uses a mixed method approach. About 389 women who participate in BIG exercise programs completed a survey. Additionally, qualitative interviews, focus groups and a photo-voice study were conducted with approximately 45 women who take part in the exercise program and those who are working in the different city-administrations to organize BIG courses.

**Results:**

15 communities were able to maintain project activities up to 18 years, while some communities ceased all activities. Effects of BIG at the structural level include low barrier exercise classes and planning groups, women only pool hours, local networks to support the project, cooking classes and social gatherings for women. The BIG-project reaches the addressed women and empowers them by increasing their PA levels, mental well-being, self-efficacy, and social network.

**Conclusions:**

BIG demonstrates that CBPR can have a long-term impact on PA promotion at the individual and structural level. The participatory approach of BIG is key to its success. By involving women in planning, it is possible to tailor all activities to their needs and thus reach them, contradicting the label that they are “hard to reach” for health promotion. Through the involvement of local stakeholders e.g. political decision makers or chairs of sports clubs it is possible to achieve change over time, however this can also make BIG vulnerable to political interests and log-rolling.

